# Laser-heating system for high-pressure X-ray diffraction at the Extreme Conditions beamline I15 at Diamond Light Source

**DOI:** 10.1107/S1600577518013383

**Published:** 2018-10-24

**Authors:** Simone Anzellini, Annette K. Kleppe, Dominik Daisenberger, Michael T. Wharmby, Ruggero Giampaoli, Silvia Boccato, Marzena A. Baron, Francesca Miozzi, Dean S. Keeble, Allan Ross, Stuart Gurney, Jon Thompson, Giles Knap, Mark Booth, Lee Hudson, Dave Hawkins, Michael J. Walter, Heribert Wilhelm

**Affiliations:** a Diamond Light Source Ltd, Harwell Science and Innovation Campus, Didcot OX11 0DE, UK; b PETRA III, Deutsches Elektronen-Synchrotron (DESY), Notkestraße 85, 22607 Hamburg, Germany; c Physics Department, Instituto Superior Tecnico (Universidade de Lisboa), Av. Rovisco Pais, Lisbon 1049-001, Portugal; d ESRF, The European Synchrotron, CS40220, Grenoble 38043, France; e Sorbonne Université, Muséum National d’Histoire Naturelle, UMR CNRS 7590, IRD, Institut de Minéralogie, de Physique des Matériaux et de Cosmochimie, IMPMC, 75005 Paris, France; f Geophysical Laboratory, Carnegie Institution for Science, 5251 Broad Branch Road NW, Washington DC, 20015, USA

**Keywords:** laser-heated diamond anvil cell, X-ray diffraction, high pressure and temperature, Diamond Light Source

## Abstract

In this article, the specification and application of the new double-sided YAG laser-heating system built on beamline I15 at Diamond Light Source are presented. This system, combined with diamond anvil cell and X-ray diffraction techniques, allows *in situ* and *ex situ* characterization of material properties at extremes of pressure and temperature. In order to demonstrate the reliability and stability of this experimental setup over a wide range of pressure and temperature, a case study was performed and the phase diagram of lead was investigated up to 80 GPa and 3300 K. The obtained results agree with previously published experimental and theoretical data, underlining the quality and reliability of the installed setup.

## Abstract 2

A double-sided, off-axis laser-heating system for monochromatic X-ray diffraction experiments using a diamond anvil cell has been installed at beamline I15 at Diamond Light Source. Measurements of the pressure–temperature phase diagram of Pb demonstrate the reliability of the setup and the high quality of the data.

## 1. Introduction   

Over the last few decades, the laser-heated diamond anvil cell (LH-DAC) coupled with *in situ* synchrotron X-ray diffraction (XRD) has become a powerful and routinely used experimental method for studying material properties under extreme conditions of pressure (*P*) and temperature (*T*). The reason why this method is taking precedence over laboratory experiments using spectroscopic probes and *ex situ* analysis is mainly because of the robustness and tunability of the components involved, nominally the LH-DAC, the synchrotron radiation facilities and the XRD technique.

The LH-DAC technique, first proposed by Ming & Bassett (1974 ▸) and widely used in the scientific community since then (Saxena *et al.*, 1995 ▸; Zerr *et al.*, 1998 ▸; Goncharov *et al.*, 2008 ▸; Boehler, 2000 ▸), takes advantage of the mechanical and optical properties of diamond, which enable studies of materials at *P*–*T* conditions in excess of 330 GPa and 5000 K (Tateno *et al.*, 2010 ▸). Diamond’s broadband transparency allows visible light (for observation and spectroscopic methods), infrared light (IR) (as heat source) and X-rays (as a probe of atomic structure) to access the high-pressure sample chamber simultaneously.

Micrometre-sized synchrotron X-ray beams are ideal for studying the structural evolution of samples of a few micrometres up to tens of micrometres in size under high *P*–*T* conditions. Furthermore, the effect of possible *P*–*T* gradients developing across the sample can be determined.

Combined with synchrotron XRD techniques, the LH-DAC is an important tool for synthesizing materials and studying their phase diagrams and thermal equation of state (EOS). Furthermore, this technique has been widely used to perform melting studies. In particular, *in situ* time-resolved investigations (seconds time-scale) of samples under extreme *P*–*T* conditions allow probing of intermediate phases, while the confinement of the sample in the LH-DAC prevents the decomposition of the final products and, most importantly, reveals detailed crystallographic information. Since the pioneering work of Shen *et al.* (2004 ▸), this technique has evolved into a routinely used and productive experimental method at synchrotron beamlines, leading to numerous major scientific advances and an expansion of high-pressure research across the disciplines of physics, chemistry, geoscience and material science (Shim *et al.*, 2004 ▸; Lavina *et al.*, 2011 ▸; Zhang *et al.*, 2014 ▸; Mao *et al.*, 2005 ▸). Several beamlines worldwide are now equipped with LH-systems for XRD-DAC experiments. Those who have reported their LH-DAC setup are ID27 (Petitgirard *et al.*, 2014 ▸) at the ESRF (France), GSECARS (Prakapenka *et al.*, 2008 ▸) and HPCAT (Meng *et al.*, 2015 ▸) at the APS (USA), 12.2.2 (Stan *et al.*, 2018 ▸) at the ALS (USA), P02.2 (Liermann *et al.*, 2015 ▸) at PETRA III (Germany) and BL10XU (Watanuki *et al.*, 2001 ▸) at SPring-8 (Japan). Some high-pressure groups have also built portable laser-heating systems that can be used in a laboratory for *ex situ* experiments or be moved to a beamline for *in situ* characterization of material properties *via* XRD or other synchrotron techniques (Kupenko *et al.*, 2012 ▸; Boehler *et al.*, 2009 ▸).

Here, we present a new state-of-the-art laser-heating system implemented at the Extreme Conditions beamline (I15) at Diamond Light Source, UK. It is the first LH-DAC–XRD facility in the UK, and complements existing resources in the field of science at extreme conditions in Europe: providing a system with exceptional stability and tunability. This new facility provides access to a large variety of different experiments, ranging from the study of thermal EOSs to the characterization of new materials, and from the determination of phase diagrams to detection of melting lines.

The article is divided into two parts. In the first part, the laser-heating system is described with all its features and components. In the second part, we report the results of our study of the phase diagram of Pb up to 80 GPa and 3300 K.

## 2. Laser heating at I15   

A state-of-the-art LH-system has been built at I15 addressing the most recent criteria necessary for a system to produce consistent and reliable results as given by Kantor *et al.* (2018 ▸) and Mezouar *et al.* (2017 ▸). Generally, the design of a LH-system for DAC experiments is defined by three main components: (i) the laser-beam delivery optics, (ii) the sample imaging and *T* measurement optics, and (iii) the coupling of the setup with the desired experimental analysis technique. The latter imposes often significant constraints on the LH-system, especially for systems to be installed at the synchrotron beamline. Specifically, the employed X-ray technique puts constraints on the components that can be used and on the space available to build the LH-system. In the following sections, the three main components of the I15 LH-system will be described in detail.

### 2.1. Laser path   

Depending on the nature of the sample to be examined, two types of lasers are commonly used for achieving high temperatures in LH-DAC experiments: (i) near-IR: Nd:YAG (Nd^3+^ doped yttrium aluminium garnet) or Nd:YLF (Nd^3+^ doped yttrium lithium fluoride) lasers (λ = 1.064 µm), and (ii) CO_2_ lasers (λ = 10.6 µm). Near-IR lasers are widely used to heat metallic and semiconducting materials (Schultz *et al.*, 2005 ▸; Shen *et al.*, 2001 ▸), whereas CO_2_ lasers are used especially for heating optically transparent minerals, oxides and organic materials (Zerr *et al.*, 1999 ▸; Salamat *et al.*, 2014 ▸).

The system built on I15 uses two 100 W fibre Nd:YAG (1.090 µm) lasers (SPI Lasers). The generally weak absorption of this near-IR light requires a very tight focusing. The small laser spot with short penetration into the material leads to large axial and radial temperature gradients. The axial thermal gradient is reduced by heating the sample from both sides. The radial thermal gradient can be minimized by slightly defocusing the lasers or by using optics to change the Gaussian profile of the laser to a flat-top profile. This allows a relatively large (up to 40 µm) uniform and homogeneous hot spot to be achieved.

**Figure 1:**
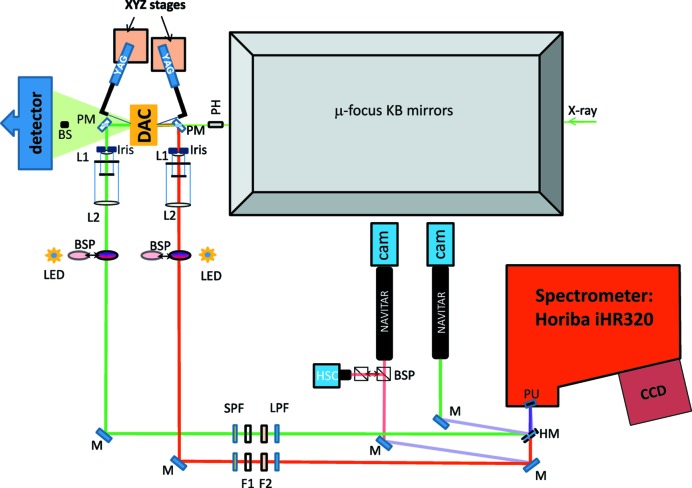
Schematic layout of the off-axis laser-heating system integrated into the micro-focus station of I15. PH: X-rays clean-up disk, PM: perforated mirror, BS: beamstop, L1: geoHEAT lens (*f* = 100 mm), L2: LotOriel lens (*f* = 1500 mm), PU: spectrometer entrance pupils, HSC: high-sensitivity camera, BSP: beam-splitter, SPF: short-pass filter, LPF: long-pass filter, F1 and F2: neutral density filters, M: mirror, CAM: CCD camera, and HM: half mirror.

For an effective LH experiment, the laser light needs to be focused on the surface of the sample and to be position-adjustable in order to heat the correct part of the sample. Both lasers can be delivered either *via* independent optics (off-axis geometry) or by sharing a common lens with the imaging part of the setup (on-axis geometry). The system built at I15 uses lasers with a TEM_00_ (Gaussian) profile to heat both sides of the sample in an off-axis geometry (see Fig. 1 ▸). The laser lights are focused on the sample surfaces using two simple plano-convex lenses with a diameter of 12.7 mm and a focal distance *f* = 50 mm (Thorlabs). Adopting an off-axis geometry allows the lasers to be defocused on the sample surfaces without affecting the image quality. In this way it is possible to achieve hot spots with uniform *T* across a region of about 40 µm in diameter (see Section 3) and without using any additional optics, such as a πShaper^®^, that might complicate the alignment procedure.

Lens tubes connected with a 12.7 mm-diameter mirror (Thorlabs) at 45° are attached to the laser fibres. These fibre-lens tube systems are mounted on motorized stages used to steer the laser light to both the upstream and the downstream side of the sample (see Fig. 1 ▸). The laser positioning is performed *via* two piezo actuators (SmarAct) with 29 and 63 mm travel range (for horizontal and focusing movements, respectively) and one linear stage (Newport) with 25 mm travel range (for vertical adjustments). Both laser delivery optics (including their stages) are placed on kinematic mounts to assure a reproducible repositioning of the laser after a sample change. The tilt, pitch and yaw of the laser delivery optic with respect to the sample position can be manually changed with a mechanical stage. These degrees of freedom provide the flexibility to tune the experimental setup and to accommodate DACs with different opening angles. The power, the position and the focus of the lasers can be remotely controlled *via* the beamline control (EPICS) and data acquisition software (GDA).

### 2.2. Sample imaging and temperature measurement   

The coupling of the sample imaging with the *T* measurement is the most critical part of the setup. In fact, the same optical components are used to view the sample and to collect the thermal radiation from the hot sample surface for *T* measurement. This constrains the geometry and the choice of optical components for the LH-system, as a high-quality and magnified image of the sample is necessary to precisely align samples that can vary in diameter from a few to hundreds of micrometres.

A magnified image of the sample can be created in different ways. Most often, a refractive optical system made up of two lenses is used because of its simplicity, flexibility, compact design, affordability and image quality at high magnification. However, at high numerical apertures, lens chromatic aberrations become important and can strongly affect the *T* measurement when refractive optical systems are used to probe hot spots with large *T* gradients (Kantor *et al.*, 2018 ▸; Mezouar *et al.*, 2017 ▸; Walter & Koga, 2004 ▸; Giampaoli *et al.*, 2018 ▸). Reflective objectives with a Schwarzschild design are an alternative (Petitgirard *et al.*, 2014 ▸; Schultz *et al.*, 2005 ▸). In this case, their pure achromatic properties lead to an aberration-free signal across the entire *T* range covered in LH-DAC experiments (1200–6000 K), at the cost of worse image quality.

Temperature metrology is of great importance in LH-DAC experiments*.* The most common way of measuring *T* *in situ* is by spectral radiometry, where the thermal-emitted radiation from the sample surface is collected with a spectrometer. The obtained intensity *I*(*λ, ∊, T*) is fitted with a Planck function in the grey body approximation,
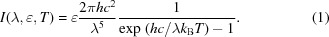
Here *h*, *k*_B_, *c* and *T* are the Planck constant, the Boltzmann constant, the speed of light and the temperature corresponding to the collected radiation, respectively; λ is the wavelength of the measured signal and ∊ is the emissivity which generally depends on pressure, temperature and wavelength. In the grey body approximation, ∊ is assumed to be independent of λ and it is used as a fitting parameter together with *T*. However, during an actual experiment, the grey body approximation is not always valid for several reasons, including aberration effects and change in emissivity caused by solid–liquid phase transitions. It is therefore of fundamental importance to know the limits of the system and to perform a critical evaluation of the *T* reading in real time.

For the LH-system installed at I15, an infinity-corrected two-lens system is used to image the sample and to collect the thermal radiation. The objectives are composed of a geoHEAT lense (πShaper), with *f* = 100 mm and a 25.4 mm opening (L1 in Fig. 1 ▸) and a plano convex lens (LotOriel) with *f* = 1500 mm and a diameter of 50.8 mm (L2). This arrangement results in a total magnification of ×15. The geoHEAT lenses have been specially designed to be apochromatic in a spectral range between 600 and 900 nm. In the present setup, a manually adjustable iris is placed on the lens (see Fig. 3) allowing the numerical aperture to be tuned between 1.75 and 0.089. In order to keep coaxial alignment and parallelism between the two lenses, they are mounted in a cage system (Thorlabs). Linear stages (Newport) with 25 mm travel range are used for the horizontal and vertical movement of each cage and for the relative movement of L1 with respect to L2 (fixed) for focusing. A set of UV enhanced Al mirrors (Thorlabs) are used to direct the focused signal into the spectrometer. A flat mirror, fabricated by the Central Laser Facility at the STFC Rutherford Appleton Laboratory, UK, with two 45 µm-diameter holes is placed at the entrance of the spectrometer. These two holes, that are vertically separated by 5 mm, act as collecting pupils for the spectrometer.

For sample imaging, the focused images are sent to two CCD MANTA cameras (Stemmer Imaging) equipped with NAVITAR objectives. Tilting the mirror at the spectrometer entrance by ∼5° allows the visualization of both sample surfaces simultaneously as proposed by Kantor *et al.* (2018 ▸). The tilt of the mirror causes the reflected beam to deviate from the principal optical axis, avoiding the use of an additional beam-splitter which would cause further aberration effects and intensity loss (see Fig. 1 ▸). The pupils at the entrance of the spectrometer are superimposed onto the sample images on the two cameras because of the system’s geometry and magnification. Each pupil (corresponding diameter of 3 µm at the sample surface) provides a spatial reference for the alignment of the lasers, the *T* reading and the X-ray position. This technique, developed by Boehler *et al.* (2009 ▸) and now used on several beamlines (Petitgirard *et al.*, 2014 ▸; Meng *et al.*, 2015 ▸; Kantor *et al.*, 2018 ▸), will be discussed in detail in the next section.

The sample can be illuminated from both sides with two ultra-bright white LEDs positioned perpendicularly to the principal optical axis (see Fig. 1 ▸). Thin-film pellicle beam-splitters are installed on a pneumatic mount. They can be moved into the optical path to send the light into the lens objective or out of the optical path for *T* measurement.

For the spectral analysis, the radiated signal from each sample surface is transmitted through the mirror pupils into a Czerny–Turner type spectrometer (Horiba iHR320, *f* = 320 mm, fitted with a Horiba 1064 × 256 pixels Synapse CCD). The incoming beams are collimated by the spectrometer’s first mirror, energy dispersed by a 150 lines mm^−1^ grating and finally focused onto two separated regions of interest (ROIs) of the CCD. The ROIs are integrated using the *LabSpec* software (Horiba) and both spectra are saved as separated *xy*-files with wavelength *versus* intensity. In order to obtain a reliable *T* measurement, the spectrometer response of the system is measured with a calibrated tungsten lamp (Walter & Koga, 2004 ▸) prior to the actual experiment. This measurement is performed for each optical configuration adopted during the experiment (iris and filters included). The final *T* is calculated by fitting a Planck function in the grey body approximation to the obtained intensity profile (normalized to the system response) using an in-house-developed software.

When carrying out laser-heating experiments, the diffuse light of the Nd:YAG laser enters both optical paths and can lead to saturation of the CCD of the spectrometer. In order to prevent this effect, short edge short-pass filters at 950 nm (Semrock, SPF, see Fig. 1 ▸) are included in the optical path to the spectrometer. In addition, to avoid second-order UV harmonics from the lasers, two 488 nm RazorEdge (Semrock) long-pass filters (LPF, see Fig. 1 ▸) are also included. Finally, two neutral density filters (Thorlabs, F1 and F2, see Fig. 1 ▸) can be moved into the optical path to attenuate the intensity of the thermal radiation, if needed. All filters can be moved remotely.

The actual *T* measurement can be severely affected by the presence of large thermal gradients, changes in the sample’s emissivity or by the presence of aberration effects caused by the refractive nature of the lenses used to image the sample. To facilitate online *T* measurements whilst monitoring the sample, a graphical software package was developed. It visualizes the raw signal together with the system response and Planck function, the linearized form of the Wien approximation of the Planck function, the sliding two-colour pyrometry and the corresponding histogram. This makes it possible to perform a critical analysis of the reliability of the obtained results in real time, as described by Benedetti & Loubeyre (2004 ▸). The software is available as standalone executable and it can also be used for post-experiment and offline analysis.

### 2.3. LH-DAC system combined with XRD   

**Figure 2:**
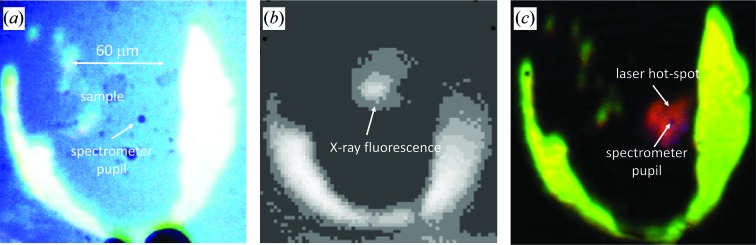
Examples of camera images collected during the alignment procedure. (*a*) Image with both transmitted and reflected light collected with the MANTA camera. The sample image and the spectrometer pupil are superimposed. (*b*) X-ray fluorescence of the pressure-transmitting medium collected with the Retiga R1 camera. The fluorescence signal is not on the spectrometer pupil because the image has been collected prior to the X-ray and image alignment. (*c*) Image of the hot spot (about 2600 K) aligned with the spectrometer pupil. All the figures are at the same scale.

**Figure 3:**
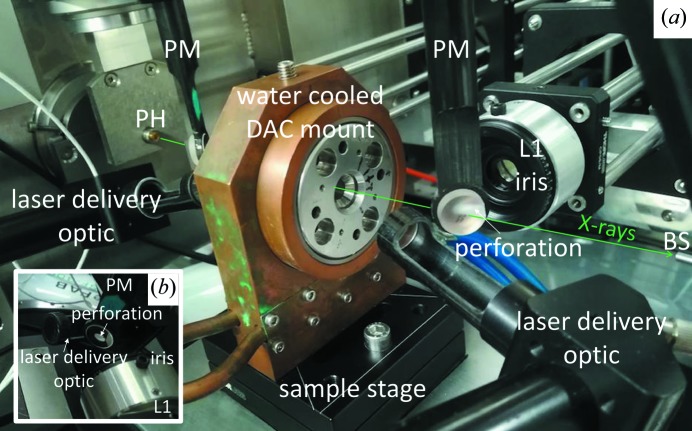
(*a*) Close-up view of the region around the pressure cell (DAC) for the off-axis laser delivery. Both laser delivery optics are visible. Perforated mirrors (PM) in the X-ray path allow the direct X-ray beam to pass through but reflect the thermal emission from the sample towards the collecting optics (only L1 visible). For spatial stability the DAC is water cooled. BS: beamstop. PH: clean-up disk. In the present picture, the iris mounted on L1 is visible. (*b*) Detailed view of the region near a perforated mirror.

When combining a LH-DAC system with an XRD setup, several factors must be considered. Firstly, at the extreme *P*–*T* conditions accessible with a LH-DAC, large *T* gradients develop across the sample and homogeneous *T* might be within an area of only 10–40 µm in diameter. Therefore, a well defined and clean X-ray beam profile with dimensions smaller than the region at homogeneous *T* is mandatory to avoid probing and collecting information from areas of the sample that are at different temperatures. For this reason, the LH-system on I15 has been built on a micro-focusing station where the X-ray beam coming from I15’s wiggler can be tuned between 20 and 80 keV and focused down to 6 µm × 4 µm [full-width at half maximum (FWHM)] by two pairs of Kirkpatrick–Baez mirrors. A clean-up disk (PH in Fig. 1 ▸) is also used to cut the tails of the X-ray beam. The alignment of the X-ray beam with the hot spot and the *T* measurement has to be precise within a micrometre or less, because of the small X-ray beam size, and is of fundamental importance for a successful and reliable *in situ* measurement. Such an alignment can be performed using the two pupils at the entrance of the spectrometer and a Retiga R1 high-sensitivity camera (HSC in Fig. 1 ▸). The pupils are superimposed on the sample image and become a spatial reference for the X-rays and laser beam (see Fig. 2 ▸ *a*). The high-sensitivity CCD camera is placed on the same path as the MANTA camera, and can be accessed with a beam-splitter (see Fig. 1 ▸) to locate the X-ray fluorescence of the pressure-transmitting medium in the DAC (see Fig. 2 ▸ *b*). The relative alignment of the X-ray image with the pupils of the spectrometer can therefore be changed by moving the lens-cage system. Finally, the laser hot spots are aligned with respect to the pupils as well (Fig. 2 ▸ *c*). While laser heating the sample, a large amount of heat is transferred to the DAC body and surrounding optics. This could lead to a progressive misalignment of the laser hot spot image with respect to the *T* measurement. For this reason the DAC is placed in a water-cooled holder (see Fig. 3 ▸) and the relative alignment of X-ray beam, hot spot and *T* measurement is checked regularly after each heating run.

The presence of the LH collecting optics in the X-ray beam path leads to an additional contribution to the diffraction pattern. In order to avoid this situation, some beamlines only perform *T* measurements on the upstream side where diffraction from the optical components is blocked by the DAC (Petitgirard *et al.*, 2014 ▸). However, this makes it impossible to detect axial thermal gradients. Other beamlines use X-ray transparent mirrors, generally made of glassy carbon (Prakapenka *et al.*, 2008 ▸; Meng *et al.*, 2015 ▸; Stan *et al.*, 2018 ▸; Liermann *et al.*, 2015 ▸), to determine the *T* measurement on both sides simultaneously. However, even though these materials are X-ray transparent, they are amorphous and therefore give a contribution to the diffraction pattern that can obscure or reduce the quality of the diffraction data, especially for weakly scattering samples.

On the present system, instead, two mirrors with a 3 mm perforation at 45° are used (see Fig. 3 ▸). The mirrors of 12.7 mm in diameter are mounted on carbon fibre holders. The perforation allows the direct X-ray beam to pass through the mirrors, avoiding any loss of flux prior to the sample and any diffraction from the downstream mirror. The only contribution to the signal on the detector is a light shadow. Thus, both sides of the sample can be visualized simultaneously while measuring the *T* and collecting diffraction data. In addition, the presence of both mirrors allows control of the shape of the hot spots on the sample surfaces and to check simultaneously their relative alignment with the spectrometer pupils during the experiment.

**Figure 4:**
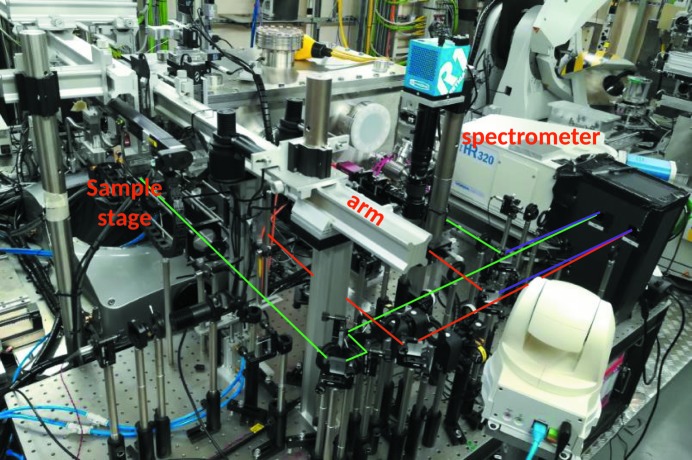
Top view of the LH-system mounted on the micro-focusing station at I15. All the optical components are mounted on a breadboard. The arm connecting the collecting optics with the sample stage is indicated. The different optical paths are highlighted by coloured lines following the notation of Fig. 1 ▸.

In order to align the optical components with the focal position of the X-rays on the sample, the LH-system has been built almost entirely on a breadboard (Fig. 4 ▸). The perforated mirrors and the two cages containing the lenses are attached to an arm mounted on the breadboard. This arm extends from the breadboard to the sample stage. In this way, the optical paths, defined by the components mounted on the breadboard (and the arm), can be aligned with the X-ray path. This is achieved by a pair of *x*, *y*, *z *stages (Huber) with 40 mm travel range, placed under the breadboard. Once the optical path is aligned (prior to the experiment), the entire LH-system can be moved to the focal spot position of the X-rays.

## 3. Application example: phase diagram of lead   

Elemental lead (Pb), one of the first metals known to man, has many applications because of its low tensile strength, high absorption of electromagnetic radiation, high resistance to corrosion and its propensity to alloy with other elements. Its behaviour under high *P* is well known. Many studies of the EOS of lead have been carried out. The high-pressure Hugoniot state (Al’tshuler *et al.*, 1960 ▸), isothermal compression (Vohra & Ruoff, 1990 ▸; Mao *et al.*, 1990 ▸), compression isentrope (Rothman *et al.*, 2005 ▸) and release isentrope (Rothman *et al.*, 2004 ▸) of Pb have all been studied. Several theoretical (Pelissier, 1984 ▸; Robinson, 2004 ▸; Mabire & Hereil, 2002 ▸; Cricchio *et al.*, 2006 ▸) and experimental (Song & Cai, 2010 ▸; Partouche-Sebban *et al.*, 2005 ▸; Godwal *et al.*, 1990 ▸) studies have focused specifically on the determination of the melting line of Pb under high *P*–*T* conditions. Different experimental techniques have been used including shock methods (Partouche-Sebban *et al.*, 2005 ▸), LH-DAC with direct visualization (speckle technique and textural analysis) of melting (Errandonea, 2010 ▸; Godwal *et al.*, 1990 ▸) and LH-DAC with XRD (Dewaele *et al.*, 2007 ▸). Initially, there was some disagreement between the results obtained using different experimental methods, with melting *T* differences varying from a few K at 20 GPa up to 500 K at 80 GPa. However, an agreement has recently been achieved between the results obtained from *ab initio* calculations (Cricchio *et al.*, 2006 ▸), shock (Partouche-Sebban *et al.*, 2005 ▸) and LH-DAC XRD experiments (Dewaele *et al.*, 2007 ▸).

Three solid phases of Pb have been observed in the *P* range from ambient to 100 GPa and a *T* range from ambient to 4000 K. Thus, this polymorphism makes Pb a suitable sample to test the reliability and limits of the I15 LH-system. In addition, as a highly scattering material, Pb also gives an intense signal from which the melting point can be extracted easily.

Membrane DACs with diamond culet sizes varying between 150 and 300 µm have been used to investigate Pb between 25 and 80 GPa. Re gaskets were prepared using spark-erosion. Samples with sizes ranging from 60 to 150 µm in diameter were cut from a 6 µm-thick Pb foil from Goodfellow and were loaded into each high-pressure chamber. Thin KCl disks (∼10 µm), FIB (focused ion beam)-cut and dried for one hour at 473 K, have been used as insulating material and as a pressure gauge during each experimental run. The X-rays were tuned to a wavelength of 0.4246 Å. The sample-to-detector (Perkin­Elmer) distance was 495 mm as determined from a LaB_6_ standard.

**Figure 5:**
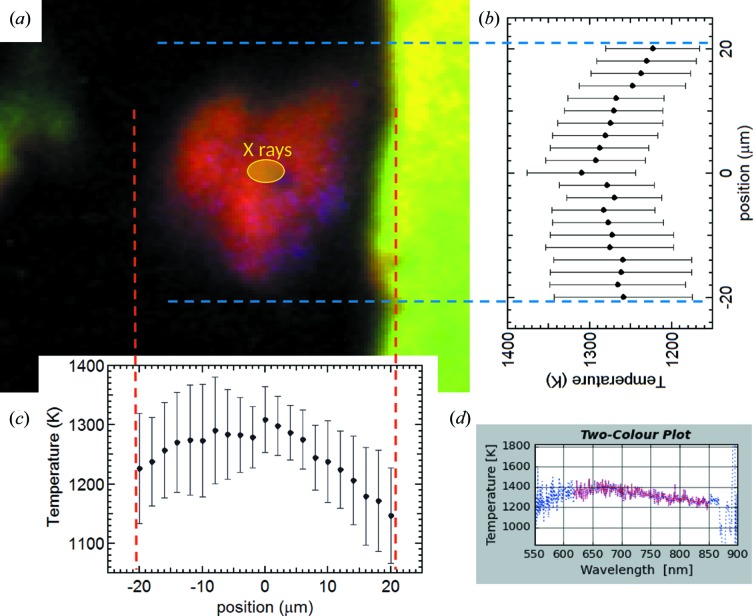
Temperature profiles of the heated Pb sample. (*a*) Image of the laser-heated hot spot overlaid with the actual X-ray beam size (6 µm × 4 µm FWHM). (*b*, *c*) Temperature scans with step size of 2 µm performed vertically and horizontally across the hot spot. The error bars have been calculated from the two-colour pyrometry according to the method described by Benedetti & Loubeyre (2004 ▸). (*d*) Example of the quality of the two-colour pyrometry obtained at the centre of the hot spot from the upstream side of the sample.

Before each laser-heating run, the sample was brought to the target *P*. The pressure was measured from the compression curve of KCl according to the thermal EOS from Dewaele *et al.* (2012 ▸)*.* The two Nd:YAG fibre lasers were individually focused on both sample surfaces and their power was linearly increased until a clear hot spot was observed on the camera. During each laser-alignment procedure, an exposure time of 4 s was used for each camera in order to be able to observe a hot spot at relatively ‘low *T*’ (around 1000 K) avoiding unwanted damage of the sample. In order to prevent the X-ray beam sampling a radial *T* gradient on the sample surface, the two lasers were defocused to maximize the size of the hot spot at uniform *T*. The two lasers were coupled together and their relative positions were tuned to obtain a uniform hot spot over about 40 µm in diameter (approximately four times bigger than the X-ray beam). Cross-scans of ±20 µm across the central position of the hot spot have been performed to check the uniformity of the *T* distribution. The scans were performed by moving, first vertically, then horizontally, the relative position of the sample image with respect to the spectrometer pupil. The results depicted in Fig. 5 ▸ reveal an even *T* distribution across a substantial part of the hot spot.

The relative alignment of the X-rays with the lasers and the spectrometer were checked before and after each heating run following the procedure described in Section 2. The power of each laser was tuned to obtain a similar *T* (*i.e.* within 50 K) on both sides. A room-temperature diffraction pattern was collected before each heating run and used as a reference during the analysis of the data collected at elevated *T*. In order to prevent any chemical reaction of the sample with the insulating material and the diamond anvils, the heating runs were performed in trigger mode. Once the power for each laser was set, the two lasers were turned on for 3 s. After a delay of 0.3 s, a diffraction pattern and a *T* measurement were collected. All diffraction patterns were collected for 2 s while the exposure time of the spectrometer varied from 2 s to 0.001 s to avoid the CCD saturation while keeping the best signal/noise ratio. Once the XRD collection ended, both laser powers were set back to zero. The triggering of the instruments involved in the data collections was performed through the beamline data acquisition software GDA.

**Figure 6:**
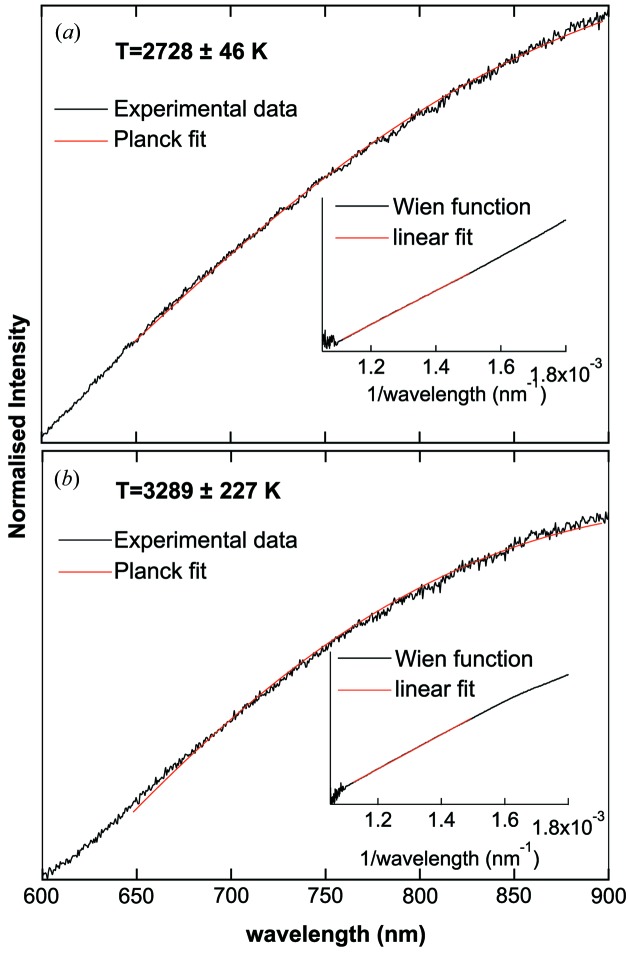
Examples of temperature measurements collected on the downstream side of the I15 LH-system while studying the phase diagram of Pb. Both graphs show the normalized intensity fitted with the Planck and the corresponding Wien function over the same region. (*a*) Signal collected at a ‘moderate’ and (*b*) at the highest *T* reached in the present experiment. The errors in the temperature measurement are given by the FWHM of the histogram of the two-colour pyrometry (not shown) as reported by Benedetti & Loubeyre (2004 ▸).

Experiments can also be performed at constant *T* or in ramp mode, *i.e.* increasing the laser power linearly from a starting to a final value, while collecting XRD and *T* data every *n* time steps. However, performing a LH experiment in trigger mode is preferable as it allows the user to check the reliability of the collected data at every step and to re-align components if needed without damaging the sample. The data analysis consisted of three steps: (i) *T* determination, (ii) extraction of crystallographic information and (iii) observation of the onset of the phase transitions. During the analysis of the *T* measurements, both the upstream and downstream *T* were determined and the average of these readings was taken to be the actual *T*. The error in each *T* measurement was assumed to be the larger value of either the difference in the upstream and downstream *T* or the FWHM of the histogram in the two-colours pyrometry (Benedetti & Loubeyre, 2004 ▸). In Fig. 6 ▸, examples of the quality of *T* measurements are shown.

The temperature information, as well as the textural and crystallographic evolution of the sample, have been analysed for every single data point. Masks to the raw diffraction images were applied on a per-image basis before they were azimuthally integrated using the processing tool in the *DIOPTAS* (Prescher & Prakapenka, 2015 ▸) suite. Diffraction data were analysed by Le Bail fitting using the routine implemented in the *TOPAS* (Coelho, 2007 ▸) software suite. Thermal *P* on the sample have been estimated from the measured volume of the B2 phase of KCl at the measured *T* using the thermal EOS of Dewaele *et al.* (2012 ▸).

**Figure 7:**
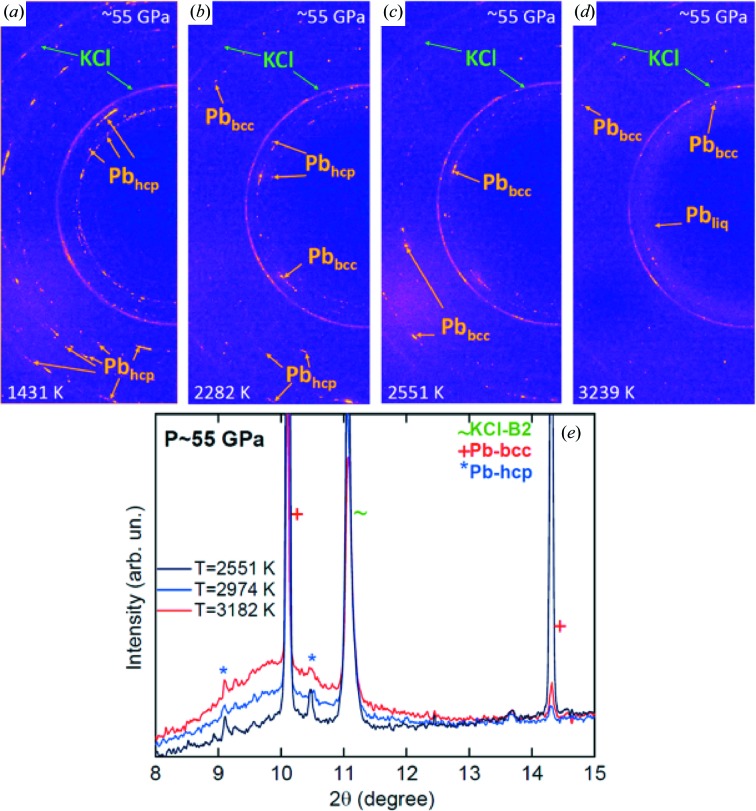
Evolution of the X-ray diffraction patterns of Pb with increasing temperature at *P* ≃ 55 GPa (λ = 0.4246 Å). The texture of the Pb sample evolves from a pure h.c.p. (*a*), *via* a coexistence region (*b*) to a b.c.c. structure (*c*). Finally, a diffuse halo (*d*) establishes the presence of molten Pb in the high-pressure chamber. (*e*) Integrated pattern before (*T =* 2551 K) and after the onset of melting (*T* ≥ 2974 K).

The obtained crystallographic data were then combined with the *T* and *P* information in order to perform an *in situ* analysis of the textural and structural evolution of the sample. Figure 7 ▸ shows an example of the textural evolution observed at ∼55 GPa as a function of *T*. The diffraction pattern changes from a powder with preferred orientation (Fig. 7 ▸ *a*) to a single crystal-like pattern during the transition from the h.c.p. (hexagonal close packed) (Fig. 7 ▸ *b*) to the b.c.c. (body centred cubic) structure (Fig. 7 ▸ *d*) and eventually to a liquid at higher *T* (Fig. 7 ▸ *d*). It is important to note that the texture of the KCl does not show major changes apart from thermal effects, preserving the insulating conditions around the sample. A thorough analysis of the onset of the phase transitions was performed by comparing several diffraction patterns before and after the observed onset. The integrated patterns in Fig. 7 ▸(*d*) show an example of the solid–liquid phase transition observed at ∼55 GPa. The intensity of the signal from the liquid increases with *T*: this is caused by the thermal gradients formed within the sample along the X-rays direction during a heating ramp. Therefore, the collected XRD signal contains contributions from the innermost regions of the sample (at lower *T*) and from the directly heated surfaces (at higher *T*) (Anzellini, 2014 ▸).

**Figure 8:**
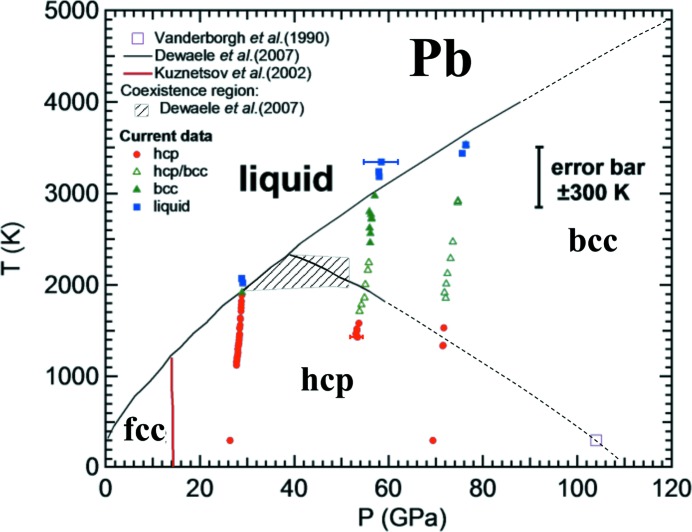
*P*–*T* phase diagram of Pb. Solid lines represent phase boundaries reported in the literature. Their extrapolations to higher pressure are shown as dashed lines. The hatched area indicates the coexistence region of h.c.p. and b.c.c. phases as reported by Dewaele *et al.* (2007 ▸). The pressure of the transition from the h.c.p. to the b.c.c. phase at ambient temperature (Vanderborgh *et al.*, 1990 ▸) has been recalibrated according to the pressure scale of Dewaele *et al.* (2004 ▸).

During the present experiment, three heating runs have been performed in a *P* range between 25 and 80 GPa and from ambient *T* to 3300 K. The obtained results are summarized in the *P*–*T* phase diagram shown in Fig. 8 ▸. The LH-system on I15 allows the collection of data at *T* as low as ∼1000 K and ramping the *T* up in steps of the order of 20–80 K. The error in each *T* measurement was calculated according to the method described above and the obtained values varied from a minimum of 10 K to a maximum of 300 K. In Fig. 8 ▸ only the maximum value of 300 K is shown for clarity reasons. The reliability of the *T* measurement also affects the error in the *P* measurement. In fact, the pressure is measured from the EOS of Dewaele *et al.* (2012 ▸) under the assumption that the temperatures of KCl and Pb are the same. However, thermal gradients are known to develop within the insulating material placed between the sample (at *T* of thousands of K) and the diamond (close to ambient *T*). Assuming a temperature of KCl at the diamond interface of 300 K, it is possible to estimate the maximum error in pressure given by the EOS of Dewaele *et al.* (2012 ▸). In particular, in this case it varies between 2 GPa at 2000 K and 4 GPa at the maximum *T* reached.

In Fig. 8 ▸, only the phase boundaries obtained in previous studies performed using the LH-DAC–XRD technique (Dewaele *et al.*, 2007 ▸; Kuznetsov *et al.*, 2002 ▸) are included for comparison (solid lines). Concerning the h.c.p. to b.c.c. phase transition, the present results are in good agreement (within the experimental errors) with the previously reported phase boundary and coexistence region. The measured solid–liquid phase boundary is also in excellent agreement with the previous results. This convincingly shows the reliability of the LH-system now available at I15.

## 4. Conclusions   

A state-of-the-art double-sided YAG laser-heating system has been built and integrated into the micro-focus station at the Extreme Conditions beamline I15 at Diamond Light Source. Laser beams are delivered off-axis and temperature measurement is made on both sides of the sample simultaneously. The newly built laser-heating system is the first laser-heating system on a synchrotron in the UK. *Ex situ* and *in situ* experiments are open for user operation. The laser-heating system offers opportunities to users from various research fields to study materials over a wide pressure and temperature range, up to 250 GPa and 6000 K. The reliability of the system has been tested and proven in a *P*–*T* range ranging from 25 to 80 GPa and 1000 K to 3300 K, by studying the phase diagram of lead.
